# Isaac McKim McCurley

**Published:** 1869-02

**Authors:** 


### OBITUARY.

Isaac McKim McCurley, D.D.S.—With sorrow we announce the death of Dr. McCurley, which occurred on Christmas day from Phthisis Pulmonalis. Dr. McCurley graduated at the Baltimore College of Dental Surgery in the Class of 1864, and the close relations we sustained with the deceased in the lecture room, infirmary, laboratory and dissecting room during two sessions, enables us to speak of him as having been a moral, studious and intelligent gentleman.

While a student, his high moral character, attention to his studies and social disposition endeared him to both Professors and Classmates, and we feel assured that no one has passed through our College Course with more respect or whose loss will be more regretted by his fellow students than the subject of this notice.

After graduating, Dr. McCurley commenced the practice of his profession in Baltimore his native city, and continued in its active duties until failing health compelled him to desist.

